# The placental lipidome of maternal antenatal depression predicts socio-emotional problems in the offspring

**DOI:** 10.1038/s41398-021-01208-x

**Published:** 2021-02-04

**Authors:** Gerard Wong, Jacquelyn M. Weir, Priti Mishra, Kevin Huynh, Brunda Nijagal, Varsha Gupta, Birit F. P. Broekman, Mary Foong-Fong Chong, Shiao-Yng Chan, Kok Hian Tan, Dedreia Tull, Malcolm McConville, Philip C. Calder, Keith M. Godfrey, Yap Seng Chong, Peter D. Gluckman, Michael J. Meaney, Peter J. Meikle, Neerja Karnani

**Affiliations:** 1 Singapore Institute for Clinical Sciences (SICS), A*STAR, Brenner Centre for Molecular Medicine, Singapore, Singapore; 2 Baker IDI Heart and Diabetes Institute, Melbourne, Australia; 3 Metabolomics Australia, Bio21 Molecular Science and Biotechnology Institute, The University of Melbourne, Melbourne, Australia; 4 Department of Psychiatry, OLVG and Amsterdam UMC, VU University, Amsterdam, the Netherlands; 5 Saw Swee Hock School of Public Health, National University of Singapore (NUS) and National University Health System (NUHS), Singapore, Singapore; 6 Department of Obstetrics and Gynaecology, Yong Loo Lin School of Medicine, National University of Singapore (NUS), Singapore, Singapore; 7 KK Women’s and Children’s Hospital, Singapore, Singapore; 8 School of Human Development and Health, Faculty of Medicine, University of Southampton, Southampton, UK; 9 NIHR Southampton Biomedical Research Centre, Southampton University Hospital NHS Foundation Trust and University of Southampton, Southampton, UK; 10 MRC Lifecourse Epidemiology Unit, University of Southampton, Southampton, UK; 11 Centre for Human Evolution, Adaptation and Disease, Liggins Institute, University of Auckland, Auckland, New Zealand; 12 Sackler Program for Epigenetics & Psychobiology at McGill University, Douglas Mental Health University Institute, McGill University, Montréal, Canada; 13 Department of Biochemistry, Yong Loo Lin School of Medicine, National University of Singapore (NUS), Singapore, Singapore

**Keywords:** Predictive markers, Molecular neuroscience

## Abstract

While maternal mental health strongly influences neurodevelopment and health in the offspring, little is known about the determinants of inter-individual variation in the mental health of mothers. Likewise, the in utero biological pathways by which variation in maternal mental health affects offspring development remain to be defined. Previous studies implicate lipids, consistent with a known influence on cognitive and emotional function, but the relevance for maternal mental health and offspring neurodevelopment is unclear. This study characterizes the placental and circulatory lipids in antenatal depression, as well as socio-emotional outcomes in the offspring. Targeted liquid chromatography-mass spectrometry covering 470 lipid species was performed on placenta from 186 women with low (*n* = 70) or high (*n* = 116) levels of antenatal depressive symptoms assessed using the Edinburgh Postnatal Depression Scale at 26 weeks’ gestation. Child socio-emotional outcomes were assessed from the Child Behavior Check List (CBCL) at 48 months. Seventeen placental lipid species showed an inverse association with antenatal EPDS scores. Specifically, lower levels of phospholipids containing LC-PUFAs: omega-3 docosapentaenoic acid (DPA), eicosapentaenoic acid (EPA), docosahexaenoic acid (DHA), and omega-6 arachidonic acid (AA) were significantly associated with depressive symptoms. Additional measurement of LC-PUFA in antenatal plasma samples at mid-gestation confirmed the reduced circulation of these specific fatty acids in mothers. Reduced concentration of the placental phospholipids also predicted poorer socio-emotional outcomes in the offspring. This study provides new insights into the role of the materno-fetal lipid cross-talk as a mechanism linking maternal mental health to that of the offspring. These findings show the potential utility of nutritional approaches among pregnant women with depressive symptoms to reduce offspring risk for later socio-emotional problems.

## Introduction

Maternal depression associates with an increased risk of anxiety and depressive disorders in the offspring^1^. Intergenerational studies (e.g., children of twins studies) emphasize the importance of non-genomic ‘shared environment’ in mediating the association between maternal depression and the risk for depression in the offspring^2^. Postnatal mechanisms that link maternal depression to impaired child development include altered parent-child interactions. However, studies examining the impact of perinatal maternal depression on the subsequent risk for depression in the offspring show strong evidence for an effect of antenatal maternal mood, independent of postnatal status^3^. Moreover, antenatal maternal depressive symptoms associate with the structure and connectivity of cortico-limbic structures that underlie cognitive—emotional functions predictive of the risk of depression^4–10^ Other reported biological associations of prenatal stress in offspring include changes in hypothalamo-pituitary-adrenal (HPA) axis and autonomous nervous systems^11^. Importantly, these effects on neural structure and connectivity as well as those on the socio-emotional development of the child are observed in community samples (i.e., normal spectrum), suggesting that the influence of maternal mood on neurodevelopment in the offspring extends across the population^12,13^.

A critical challenge for prevention is that of identifying the relevant intrauterine mechanisms through which maternal mood influences the development and health of the offspring. The availability of specific nutrients, including lipids, are critical for fetal neurodevelopment^14^, and maternal depression associates with altered food intake and circulating levels of multiple nutrients, including B vitamins and polyunsaturated fatty acids^15,16^. Specific maternal micronutrient deficiencies like folate insufficiency have been associated with a reduction in brain volume and poorer cognitive outcomes in offspring^17^. Since the fetus is dependent upon the maternal supply of essential nutrients, the nutritional status of the mother may directly influence that of the fetus, with downstream effects on neurodevelopment and mental health. The potential combined effects of depression and nutrition, whereby stress leads to unhealthy eating patterns during pregnancy have been highlighted in previous studies^18–20^. Lower neurodevelopmental scores in children have been reported as a consequence of the combinatorial effects of maternal stress and low dietary omega-3:omega-6 ratio^21^. An increase in the levels of pro-inflammatory cytokines in women due to interaction of stress and high-fat meal consumption^22^ can affect neurodevelopmental outcomes in the offspring^23–26^. Studies of associations between maternal depressive symptoms and nutrient status commonly focus on measuring nutrient intake in the postnatal period. Increasing evidence for the importance of antenatal maternal mood on fetal neurodevelopment underscores the potential importance of antenatal maternal-fetal nutrition. A critical gap in the literature derives from the reliance on measures of dietary intake in the absence of direct measures of nutrient status. Importantly, measurements of maternal nutrient intake may not accurately assess its availability for fetal development and reflect consequent impact. Nutrient delivery to the fetus is regulated by multiple processes, including maternal metabolism and transport across the placenta. Finally, while there is compelling evidence for the association between antenatal maternal depression and socio-emotional outcomes in the offspring, the relevance in utero signal is unknown, which is a major obstacle for identifying effective interventions.

Our study characterizes the placental and circulatory lipids in relation to antenatal depression. The longitudinal design not only identifies the specific lipid alterations in maternal circulation and placenta as a function of mental health status, but also its downstream associations with socio-emotional outcomes in the offspring. We thus provide new insights into the role of the maternal-fetal lipid cross-talk as a mechanism linking maternal mental health to that of the offspring.

## Methods

### Study design and participants

Growing Up in Singapore Towards healthy Outcomes (GUSTO)^16^ is a prospective birth cohort study involving deep phenotyping of pregnant women and their offspring^16^. Written informed consent was obtained from all study participants. Soh et al.^16^ provides a detailed description of the GUSTO project. Briefly, the GUSTO study was designed to investigate the developmental origins of individual differences in mutliple health outcomes. Pregnant women in their first trimester and of ≥18 years of age were recruited from the two major public hospitals in Singapore [KK Women’s and Children’s Hospital (KKH) and the National University Hospital (NUH)]. This study was ethically approved by the Domain-Specific Review Board of NUH and the Centralized Institutional Review Board of KKH. Eligible participants had to hold Singapore citizenship or permanent residency, or intent to reside in Singapore for the next five years, were of Asian ethnicity, and willing to contribute biosamples. Of the 1247 recruited pregnant women in GUSTO, 1048 had placenta collected at delivery. All available placenta samples underwent targeted lipidomic analysis from which we selected subjects (*N* = 70) of Chinese and Malay ethnicity with low levels of depressive symptoms (EPDS scores of <2) and subjects (*N* = 116) with high levels of depressive symptoms (EPDS scores in the subclinical/clinical range of depression of more than 10) at 26–28 weeks gestation. Maternal age (*t*-test), ethnic composition (chi-squared test), parity (Wilcoxon), pre-conception BMI (*t*-test) were not statistically significant between the groups of subjects (Table S1). Pregnancies conceived by in vitro fertilization and multiple pregnancies were excluded from this study.

### Psychological assessments

The Edinburgh Postnatal Depression Scale (EPDS) questionnaire was administered to mothers at 26 weeks of pregnancy to quantify levels of maternal depressive symptomatology. The EPDS is a widely used 10-item self-report scale designed as a screening instrument for postnatal depression validated for use in prenatal and postnatal depression^27^. Each item of the EPDS was scored on a four-point scale (0–3). The reliability of the EPDS score was 0.82 assessed using Cronbach’s analysis for our cohort. Higher EPDS scores indicate a greater number of depressive symptoms.

When children were 48 months of age, parents completed the Child Behavior Checklist (CBCL/1.5–5), a parents’ questionnaire that contains 99 problem items rating various scales reflecting dimensions of child socio-emotional function spanning categories of emotionally reactive, anxious/depressed, somatic complaints, withdrawn, sleep problems, attention problems, aggressive behavior, internalizing problems, externalizing problems and five DSM-oriented scales namely DSM anxiety problems, DSM affective problems, DSM pervasive developmental problems, DSM oppositional defiant problems and DSM attention-deficit/hyperactivity disorder (ADHD). The CBCL was validated and showed high reliability in Singaporean children^28^. Previous studies show that antenatal maternal depressive symptoms associates with internalising scores in children in community samples^12^.

### Lipidomics of placental samples

Maternal and fetal-facing sides of the placenta were collected, stored and homogenized as detailed under supplemental methods together with a detailed description of lipid extraction and analysis methods^29,30^. Briefly, lipids were extracted from 20 µL (~100 μg of protein) of placental homogenate using chloroform/methanol (2:1, 20 volumes). Lipid analysis was performed by liquid chromatography, electrospray ionisation-tandem mass spectrometry using an Agilent 1290 liquid chromatography system with a 50 mm Zorbax Eclipse Plus 1.8 µm C18 column, combined with an Agilent 6490 triple quadrupole mass spectrometer. The relative concentration of each lipid species was calculated from the area of the resultant chromatograms for the lipid species and the corresponding internal standards. A pilot study consisting of 57 maternal-facing placenta samples and 57 fetal-facing placenta samples were run initially to assess potential biases in lipidomic profiles arising from different sampling positions. 1048 maternal-facing placenta samples were subsequently analysed across 7 batches in the main experimental run. The various lipid classes/subclasses profiled have been tabulated in Table S2.

### Analysis of fatty acids in plasma phosphatidylcholine

Maternal fasting blood samples collected at 26–28 weeks’ gestation were processed within 4 h and stored at −80 °C. Plasma lipids were extracted using chloroform–methanol (2:1, v/v), and phosphatidylcholine (PC) was isolated by solid-phase extraction (details provided under Supplemental methods). Fatty acid methyl esters (FAME) were generated from PC after reaction with methanol containing 2% (v/v) sulfuric acid, extracted into hexane and separated by gas chromatography (GC). FAME were identified by comparison with retention times of previous standard runs and quantified using ChemStation software (Agilent Technologies). The absolute fatty acid concentration measurements were expressed as µg/mL of plasma. The profiled plasma fatty acids included myristic acid, palmitic acid, palmitoleic acid, stearic acid, oleic acid, cis vaccenic acid, linoleic acid, C18:3(n-6), C20:0, C20:1(n-9), C20:2(n-6), C20:3(n-6), arachidonic acid, C22:0, C20:4(n-3), C22:4(n-6), EPA, DPA, DHA, C18:3(n-3).

### Statistical analysis

In the pilot study, 57 maternal-facing placenta samples and 57 fetal-facing placenta samples were analysed in a single batch. Pooled placenta quality controls (QCs) were inserted in the run once every 10 study samples. The percentage coefficient of variation was computed on the basis of the QCs for all lipid species. Lipid species with a %CV of more than 50% were omitted from subsequent analysis. A polynomial-fit (order 7) correction based on QCs was employed to minimize the effects of drifts in sensitivity in the mass spectrometer over the batch, this approach is similar to the QC-RLSC protocol that is described in Dunn et al.^31^. Multiple linear regression (see Model 1 in the Supplemental statistical methods) was used to determine the association of placenta lipid species with antenatal EPDS scores. Linear regression was run separately for the maternal-facing placenta samples and the fetal-facing placenta samples and the beta coefficients from both analyses were compared using Pearson’s correlation (see Fig. S1).

In the main placenta experiment, 1048 maternal-facing placenta samples were analysed in targeted lipidomic analysis run across 7 batches. Pooled placenta QCs were also inserted once every 10 study samples in the run. The percentage coefficient of variation was computed on the basis of the QCs for all lipid species (see Fig. S2). Lipid species with a %CV of more than 50% were omitted from subsequent analysis. Within each batch, a polynomial-fit (order 7) correction based on QCs was employed to minimize the effects of drifts in sensitivity in the mass spectrometer over the course of the run. After the application of the polynomial-fit correction, the measurements of the lipid species in all samples of each batch were aligned by multiplying the lipid levels by the reciprocal of the ratio of the median lipid measurement in the QCs of each batch to the median level of the lipid in the QCs across all batches, this approach is also commonly known as median centering. A PCA visualization of the raw data and the batch-corrected data can be found in the Supplementary material (see Fig. S3). Lipid species with a correlation coefficient of >0.4 or <−0.4 with respect to the run order were deemed to have residual batch effects and were omitted from subsequent analysis. 470 lipid species passed quality filtering and were analysed in downstream statistical analysis.

Multiple adjusted linear regression was used to determine (i) the association of placental lipids with antenatal EPDS, (ii) the association of plasma phosphatidylcholine fatty acids with antenatal EPDS (iii) association of antenatal EPDS with CBCL scales in the offspring at 48 months and (iv) the association of antenatal depressive mood related placental lipids with CBCL scales in the offspring at 48 months. FDR-adjusted *p*-values with a cutoff of 0.05 were used to identify the significant candidate lipids associated with antenatal depression. Placental lipid species and plasma fatty acids were log10-transformed and *z*-score standardized before incorporation into the model. CBCL scores were utilized in their t-score format. Covariates such as pre-pregnancy BMI were also log10-transformed and *z*-score standardized before use. Missing values for fish-oil consumption were mode imputed while missing values for highest education age were mean imputed according to highest education level. The details of each specific model can be found in the Supplementary material.

## Results

### High correlation of lipid profiles between maternal and fetal sides of placenta

In a pilot study (*N* = 57) comparing the standardized beta-coefficients of linear regression analysis based on the maternal and fetal-facing placenta (Fig. S1 in the Supplementary material) we found that the measurements of lipids in both sets of samples were highly correlated (rho = 0.843, *p* = 3e−129) and that measurements of the maternal-facing placenta samples would be sufficiently representative of the shared placental environment between the mother and offspring.

### Antenatal depression associates with distinct lipid profile of placenta

In the main lipidomics study we analysed placenta from 186 women with low (*n* = 70, EPDS score <2), or high (*n* = 116, EPDS score >10) levels of antenatal depressive symptoms at 26 weeks gestation. We selected the EPDS cut-off based on an extensive validation analysis that designated antenatal scores of 10–13 as reflecting probable mild depression with higher scores indicating a greater level of depression^32^. The multiple linear regression analysis, identified 17 lipid species in placenta (Table 1) to be significantly (FDR-corrected *p* < 0.05) associated with EPDS scores. These lipid species included phosphatidylcholine (PC), phosphatidylcholine plasmalogen (PC(P)), phosphatidylethanolamine (PE), phosphatidylethanolamine plasmalogen (PE(P)), alkylphosphatidylethanolamine (PE(O)), phosphatidylinositol (PI), phosphatidylserine (PS) and sphingomyelin (SM) with the majority of lipid species (94%) being phospholipids. There was a distinct preponderance for phospholipids containing LC-PUFAs i.e., EPA (C20:5, n-3), DPA (C22:5, n-3), DHA (C22:6, n-3) or AA (C20:4, n-6). 76% of the significant lipid species contained at least one form of the stated LC-PUFAs, suggesting specificity in the associations of phospholipids containing LC-PUFAs with antenatal depressive symptoms. The results from the linear regression model used above also accounted for the confounding from the use of fish-oil supplements. Findings from the model unadjusted for fish-oil supplementation are summarized under Table S3.

**Table 1:** List of placental lipids significantly associated (FDR *p* < 0.05) with EPDS at 26 weeks as determined by multiple adjusted linear regression.

Placental lipids	Fatty acid composition	Beta coefficient (standardised)	Lower bound	Upper bound	FDR-corrected *p*
PE(P-17:0/20:4)(a)	PE(P-17:0/20:4,n-6)	−0.587	−0.873	−0.302	0.024
PE(P-17:0/22:6)(a)	PE(P-17:0/22:6,n-3)	−0.569	−0.850	−0.289	0.024
PE(O-36:5)	PE(O-16:1/20:4,n-6), PE(O-16:0/20:5,n-3)	−0.540	−0.825	−0.256	0.041
PC 35:5	PC(15:0/20:5,n-3)	−0.499	−0.775	−0.222	0.046
PE(P-16:0/20:5)	PE(P-16:0/20:5,n-3)	−0.503	−0.784	−0.222	0.046
PC 37:6	PC(15:0/22:6,n-3)	−0.495	−0.775	−0.215	0.046
PC 34:5	PC(P-14:0/20:5,n-3)	−0.483	−0.762	−0.204	0.046
PS 40:5	PS(18:0/22:5,n-3)	−0.497	−0.787	−0.207	0.046
PE(P-18:0/20:5)	PE(P-18:0/20:5,n-3)	−0.480	−0.761	−0.200	0.046
PE 40:5(a)	PE(18:0/22:5,n-3)	−0.487	−0.776	−0.198	0.046
PI 35:2	PI(17:0/18:2)	−0.484	−0.771	−0.197	0.046
SM 35:1(b)	SM(17:1/18:0)	−0.471	−0.753	−0.188	0.046
PC 33:2	PC(15:0/18:2)	−0.464	−0.744	−0.184	0.046
PE(P-16:0/22:5)(a)	PE(P-16:0/22:5,n-3)	−0.479	−0.769	−0.189	0.046
PC(P-36:5)	PC(P-16:0/20:5,n-3)	−0.464	−0.745	−0.182	0.046
PC 39:5(a)	PC(17:0/22:5,n-3)	−0.465	−0.750	−0.180	0.046
PI 40:5(a)	PI(18:0/22:5,n-3)	−0.464	−0.749	−0.179	0.046

### Omega fatty acids levels are longitudinally associated with antenatal depressive symptoms

To determine if the placental lipid signatures were also observed at an earlier timepoint in pregnancy (26 weeks) we assessed a selection of fatty acids in maternal plasma and identified three fatty acids in antenatal plasma phosphatidylcholine to be associated with antenatal depressive symptoms at 26 weeks. DPA (beta = −0.469, *p* = 0.001) and EPA (beta = −0.389, *p* = 0.005) were inversely associated with antenatal depressive symptoms while dihomo-gamma-linolenic acid (C20:3, n-6) or DGLA (beta = 0.331, *p* = 0.026) was positively associated with antenatal depressive symptoms. DHA (beta = −0.213, *p* = 0.154) and AA (beta = 0.121, *p* = 0.43) in antenatal plasma were not significantly associated with antenatal depressive symptoms, although the direction of association of DHA with antenatal depressive symptoms was consistent with that of DPA and EPA (Table 2).

**Table 2:** Cross-sectional association of fatty acids in plasma phosphatidylcholine with EPDS at 26 weeks as determined by multiple adjusted linear regression.

Fatty acids in plasma phosphatidylcholine	Beta coefficient (standardised)	Lower bound	Upper bound	*p*
C22:5(n-3)	−0.469	−0.744	−0.195	0.001
C20:5(n-3)	−0.389	−0.660	−0.118	0.005
C20:3(n-6)	0.331	0.041	0.620	0.026
C16:1(n-7)	0.295	−0.001	0.592	0.052
C22:4(n-6)	−0.266	−0.561	0.030	0.079
C18:1(n-9)	0.265	−0.031	0.562	0.081
C22:6(n-3)	−0.213	−0.504	0.079	0.154
C20:0	−0.209	−0.505	0.087	0.168
C18:3(n-6)	−0.156	−0.455	0.143	0.308
C20:1(n-9)	−0.135	−0.434	0.163	0.376
C20:4(n-6)	0.121	−0.178	0.420	0.430
C18:3(n-3)	−0.106	−0.404	0.191	0.484
C18:0	−0.101	−0.398	0.195	0.504
C22:0	−0.091	−0.390	0.208	0.551
C20:2(n-6)	0.061	−0.232	0.354	0.683
C18:2(n-6)	−0.050	−0.350	0.249	0.743
C20:4(n-3)	−0.036	−0.328	0.255	0.808
C16:0	−0.030	−0.325	0.266	0.845
C18:1(n-7)	−0.016	−0.313	0.282	0.918
C14:0	0.003	−0.295	0.300	0.985

### Antenatal depressive symptoms and child socio-emotional problems

As expected multiple linear regression analysis revealed significant associations (*p* < 0.05) between antenatal EPDS scores and 13 out of the 15 CBCL scales (Table 3). The associations were all positive implying that higher EPDS scores are associated with increased socio-emotional problems in the offspring. Only DSM Oppositional Defiant Problems and DSM ADHD scales were not significantly associated with antenatal EPDS scores likely due to the age of the children, which pre-dates the age for these clinical outcomes.

**Table 3:** Association of antenatal EPDS at 26 weeks with CBCL scales at 48 months in the offspring as determined by multiple adjusted linear regression.

CBCL scales (*t*-scores)	Beta coefficient	Lower bound	Upper bound	*p*
DSM anxiety problems	6.44	3.61	9.28	0.00002
Internalizing problems^‡^	9.09	4.95	13.23	0.00004
Total problems	8.68	4.56	12.80	0.0001
Anxious/depressed^a^	4.67	2.14	7.20	0.0004
Emotionally reactive^a^	4.28	1.81	6.75	0.001
Externalizing problems^‡^	6.11	2.19	10.02	0.003
DSM affective problems	4.18	1.43	6.94	0.004
Sleep problems^‡^	3.50	1.14	5.86	0.004
DSM pervasive developmental problems	4.01	0.95	7.08	0.012
Somatic complaints^a^	3.37	0.67	6.06	0.016
Attention problems^b^	2.81	0.51	5.11	0.018
Aggressive behavior^b^	2.44	0.26	4.61	0.030
Withdrawn^a^	3.62	0.36	6.87	0.031
DSM oppositional Defiant problems	1.25	−0.86	3.36	0.249
DSM ADHD	0.92	−1.09	2.92	0.373

^a^Components of internalising problems score.

^b^Components of externalising problems score.

^‡^Broad-Band Scales.

### Placental lipids linked with antenatal maternal depression are also associated with child behavioral outcomes

**Fig. 1:**
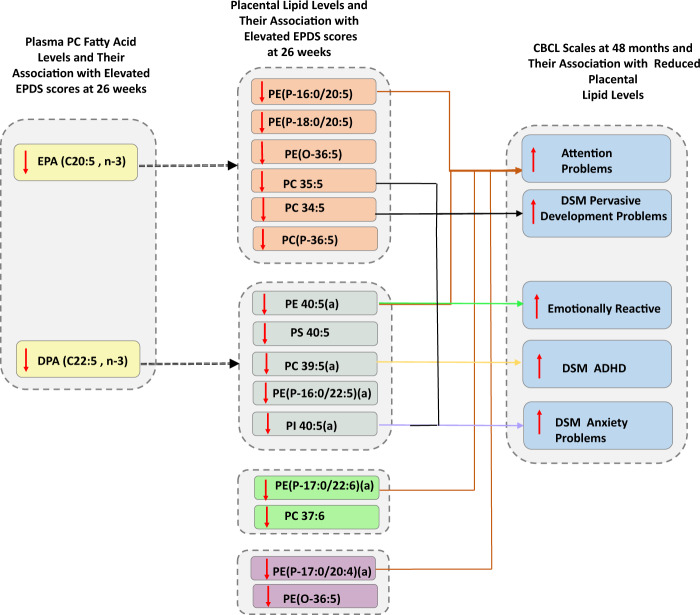
Schematic summary of lipids linked with antenatal maternal depression and child socio-emotional outcomes at 48 months. First panel represents the directionality of association of antenatal plasma n-3 fatty acids with elevated EPDS scores. Second panel represents the directionality of association of placental lipid levels with elevated EPDS scores. Third panel represents the directionality of association between CBCL scales and placental lipids. Up arrows imply positive association, while down arrows imply inverse association.

We next assessed the association of 17 antenatal depression-linked lipid species with CBCL scores at 48 months and identified 8 lipids showing an association (*p* < 0.05) with five CBCL scales (Table 4). These associations persisted after adjusting for maternal EPDS status suggesting a direct association between the lipid and the socio-emotional outcome. PE 40:5(a), PE(P-16:0/20:5), PE(P-17:0/20:4)(a), PE(P-17:0/22:6)(a) were significantly associated with the Attention Problems scale, PC 34:5 PC 35:5 and PI 40:5(a) were significantly associated with the DSM Pervasive Developmental Problems scale, PE 40:5(a) with the Emotionally Reactive scale, PI 40:5(a) with DSM Anxiety Problems scale, and PC 39:5(a) with DSM ADHD scale. PI 40:5(a), which contains DPA, was particularly noteworthy as it was associated with all the analysed CBCL scales at either the significance (*p* ≤ 0.05) or trend (*p* > 0.05 < 0.1) level. EPA and DPA contributed to the fatty acid composition of three significant lipid species each while DHA and AA contributed to the fatty acid composition of one significant lipid species each (summarized in Table 4). Tables S4 and S5 in the Supplementary material summarises the linear regression results for all 15 CBCL scales without including EPDS as a covariate. The main findings from this study have been summarised in a schematic in Fig. 1.

**Table 4:** Association of placental lipid levels with CBCL scales at 48 months in the offspring as determined by multiple adjusted linear regression.

Placental lipids	Attention problems		DSM pervasive developmental problems		Emotionally reactive		DSM anxiety problems		DSM ADHD	
	Beta	*p*	Beta	*p*	Beta	*p*	Beta	*p*	Beta	*p*
PC 33:2	−1.128	0.058	−1.006	0.207	−0.738	0.251	−1.070	0.146	−0.908	0.081
**PC 34:5**^a^	−0.004	0.995	**−1.640**	**0.039**	−0.854	0.185	−1.223	0.097	−0.155	0.768
**PC 35:5**^a^	−0.657	0.290	**−1.781**	**0.030**	−0.918	0.168	−1.396	0.067	−0.661	0.222
PC 37:6	−1.079	0.069	−1.414	0.073	−0.956	0.135	−1.414	0.053	−0.917	0.076
**PC 39:5(a)**^b^	−1.137	0.054	−1.096	0.164	−1.046	0.099	−1.086	0.136	**−1.039**	**0.043**
PC(P-36:5)	−0.446	0.445	−0.983	0.206	−0.368	0.558	−0.553	0.443	−0.446	0.381
**PE 40:5(a)**^b^	**−1.672**	**0.004**	−1.153	0.147	**−1.405**	**0.027**	−1.330	0.070	−0.922	0.075
PE(O-36:5)	0.005	0.994	0.875	0.265	0.573	0.366	0.951	0.190	0.095	0.854
**PE(P-16:0/20:5)**^a^	**−1.268**	**0.031**	−0.901	0.253	−1.099	0.083	−0.520	0.477	−0.978	0.057
PE(P-16:0/22:5)(a)	−0.918	0.116	−0.070	0.929	−0.699	0.267	−0.619	0.392	−0.403	0.431
**PE(P-17:0/20:4)(a)**^c^	**−1.422**	**0.020**	−1.434	0.081	−0.341	0.609	0.053	0.945	−0.769	0.154
**PE(P-17:0/22:6)(a)**^d^	**−1.250**	**0.038**	−1.101	0.173	−0.664	0.310	−0.539	0.472	−0.876	0.097
PE(P-18:0/20:5)	−0.436	0.466	−0.896	0.260	−0.604	0.347	−0.589	0.424	−0.448	0.390
PI 35:2	−0.649	0.275	−0.295	0.710	−0.072	0.910	−0.398	0.588	−0.623	0.229
**PI 40:5(a)**^b^	−1.145	0.052	**−1.550**	**0.048**	−1.206	0.057	**−1.611**	**0.026**	−0.905	0.078
PS 40:5	−0.972	0.103	−1.014	0.202	−0.564	0.381	−0.846	0.251	−0.470	0.367
SM 35:1(b)	−0.450	0.449	−0.975	0.217	−0.032	0.961	−0.741	0.311	−0.996	0.053

^a^Contains EPA (C20:5, n-3).

^b^Contains DPA (C22:5, n-3).

^c^Contains AA (C20:4, n-6).

^d^Contains DHA (C22:6, n-3).

Bold values represent significant associations (*p* < 0.05).

## Discussion

We analysed the placental lipidome of 186 subjects with differing levels of depressive symptoms assessed at 26 weeks of pregnancy. A total of 470 lipid species in the placenta were considered in statistical analysis. Phospholipids such as PC, PE, and PI containing polyunsaturated fatty acids like DHA, DPA, EPA, and AA had the strongest associations with antenatal depressive symptoms. A cross-sectional association analysis of the second trimester (26 weeks) maternal plasma phosphatidylcholine fatty acids and EPDS replicated the placental findings for EPA and DPA. For DHA, the direction of association with EPDS was the same between placenta and plasma, though the association passed significance only in the placenta.

Mammalian cells are reported to contain ~1000–2000 lipid species involved in various cellular functions, including neuronal signaling. A large number of lipids in the plasma membrane regulate the membrane function as a barrier between the intracellular and extracellular spaces. Membrane lipids also determine the localization and function of proteins within the membrane to regulate the synaptic throughput. Walther et al.^33^ proposed a glucocorticoid positive spiral pathway to explain the biological mechanism of depression-induced alteration in lipid profiles. In this pathway, a stress-induced increase in levels of glucocorticoids leads to higher phospholipase D activity causing enhanced conversion of PC and PE lipid classes to phosphatidic acid, lysophosphatidylcholine, and lysophosphatidylethanolamine. Phosphatidic acid further converts to diacylglycerols and together with lysophosphatidylcholine and lysophosphatidylethanolamine, lead to membrane bending and increased influx of glucocorticoids into the cell, thereby forming a spiral pathway. There are alternatives. Depression may lead to heightened oxidative stress and lowered anti-oxidant defences leading to increased lipid peroxidation and higher levels of peroxidation products such as malondialdehydes^34^.

There is also compelling evidence on the critical role played by PUFAs in brain development. The most abundant PUFA in the brain includes ARA (n-6) and DHA (n-3), accounting for about one fifth of the brain’s dry weight. The effects of omega-3 fatty acid deprivation on brain content and behavior can accumulate over life-course and over several generations. In adults, DHA and DPA can be synthesised from linoleinic acid (18:3), but are primarily derived from the diet. The placenta and fetus lack the desaturase enzymes required to synthesize the LC-PUFAs and are therefore entirely reliant on the maternal supply^35^, thus LC-PUFAs are considered essential fatty acids for the fetus. During pregnancy, the essential fatty acids in the maternal circulation available for transplacental transfer to the fetus are derived from the maternal diet, from maternal metabolism of precursor fatty acids and the breakdown of adipose tissue stores. The importance of the association of these LC-PUFAs with antenatal maternal depressive symptoms is apparent in the finding that the level of circulating phosphatidylcholine containing LC-PUFAs in the mother is strongly associated with those in the placenta. This is consistent with the general notion that the concentration of maternal circulating free fatty acids is a main determining factor of the umbilical cord free fatty acid concentration in uncomplicated pregnancies^36^. Although not all phospholipids were profiled in antenatal maternal plasma, phosphatidylcholine, the most abundant phospholipid class provides a reasonable gauge of the LC-PUFAs levels in plasma phospholipids.

Fetal free fatty acid levels are dependent upon placental transfer primarily determined by maternal diet and important for neurodevelopment. Observational studies suggest that consumption of fish rich in omega-3 LC-PUFAs during pregnancy is positively associated with neurodevelopmental outcomes, including child behavioral outcomes^37–40^. Barker et al.^8^ showed that an association between antenatal maternal depression and child neurodevelopmental outcomes was mediated by a healthy maternal diet that included sources of omega-3 and 6 fatty acids.

We used a prospective longitudinal study design to determine if maternal circulating lipids, antenatal EPDS scores together with the placental lipid profile predict neurodevelopmental outcomes in the offspring, focusing on measures of socio-emotional problems that are known to associate with antenatal maternal depression and predict the later risk for psychopathology in the child^12,41^. We identified that lower levels of phospholipid species containing EPA, DPA, DHA, and AA fatty acids in the placenta were linked to reduced levels of EPA, DPA in plasma phosphatidylcholine during pregnancy and that these phospholipids were significantly associated with poorer socio-emotional outcomes on multiple CBCL scales at 48 months of age.

The LC-PUFAs that drive the association with CBCL scales are omega-3 EPA (20:5, n-3), DPA (22:5, n-3), DHA (22:6, n-3) and omega-6 arachidonic acid (20:4, n-6). These findings are consistent with those using measures of dietary reports documenting a positive association between LC-PUFA intakes and better neurodevelopmental outcomes^42^. Our findings suggest that antenatal maternal nutritional status may serve as an in utero factor that drives the association between maternal depressive symptoms and neurodevelopment outcomes in the offspring, including phenotypes that predict the later risk for depression. This conclusion is consistent with the premise that fetal nutrition shapes fetal neurodevelopment and later mental health outcomes^43^. There is evidence from developmental neuroscience for the biological plausibility of an association between fetal lipids and neurodevelopment. Fatty acids are required for optimal growth, development, and function of brain tissue^14^. The third trimester of pregnancy features dynamic structural neural growth of the fetal brain, which coincides with a massive increase in the selective accumulation of DHA and other LC-PUFAs, which are preferentially taken up and transferred across the placenta^35^. Studies with model systems show that perinatal brain DHA accrual is required for normal neurotrophic factor expression, neurite outgrowth, neurogenesis and migration and neuronal differentiation and the function of neurotransmitter systems as well as transmitter receptor function through influences on membrane fluidity^44–46^.

These findings suggest that an in utero nutritional status deficient in placental phospholipids containing LC-PUFAs, relates to both antenatal maternal depressive symptoms and later childhood socio-emotional problems. Our study benefits from a prospective, longitudinal design with direct measures of maternal nutrient status and placental lipidomic profiling. We postulate that a low maternal omega-3 level may limit the ability of the expecting woman to cope with prevailing psychological stressors leading to antenatal depressive symptoms during pregnancy, whilst at the same time being unable to meet the demands of the fetus who is competing for the limited LC-PUFA resource, resulting in suboptimal fetal brain development and subsequently poor child metal health outcomes.

A limitation of our study design is in the reliance on the EPDS, rather than direct clinical assessment of depression; we therefore refer to the findings as reflecting an association between the number of depressive symptoms and lipidomic profiles. However, the EPDS is a highly validated screening tool for perinatal depression^25^. Importantly, our sample included women with EPDS scores largely falling in the high range of sub-clinical to lower range of clinical scores, and were thus not unique to more severe cases of maternal depression. Likewise, our previous neuroimaging studies suggest that the number of depressive symptoms associates with fetal cortico-limbic development and that this association is observed in non-clinical community samples, suggesting that maternal mental health influences fetal neurodevelopment across the population^4,5,13^. Similarly, while CBCL scores originate from parental questionnaires rather than formal neurodevelopmental assessments they are validated and showed high reliability in Singaporean children^28^. The objective measure of placental lipids is a major strength of our study, however further analysis of cord blood can provide a more accurate quantification of true transplacental transfer of lipids.

This study provides new insights into the biological pathways by which maternal emotional wellbeing influences fetal neurodevelopment and the later risk for psychopathology. Our results suggest a potential utility of nutritional approaches in the prevention of risk for later offspring psychopathology among pregnant women with high levels of depressive symptoms. This is particularly pertinent in light of the reluctance of pregnant women to adopt pharmacological interventions, including anti-depressant medications. We note, however, that while diet is inevitably important in determining fatty acid availability for the fetus, there might also be influences of genetic variations that affect the free fatty acid synthesis, metabolism, and transport across the placenta that need to be considered in tandem with nutritional supplementation efforts.

## Supplementary information

Supplementary material

## Data Availability

Statistical analyses were conducted using scripts and libraries written in MATLAB® R2017a and R-3.6.3. Codes used for the analyses in this work are available upon request.
