# Supplementary material for: The placental lipidome of maternal antenatal depression predicts socio-emotional problems in the offspring
Source: Transl Psychiatry. 2021 Feb 4;11:107. doi: 10.1038/s41398-021-01208-x (PMC7862650; doi:10.1038/s41398-021-01208-x)
Supplement: Supplementary file 1 — Supplementary material [file 41398_2021_1208_MOESM1_ESM.docx]

**Supplementary Material**

Figure S1. Scatterplot comparing beta-coefficients of 57 fetal-facing placenta and corresponding maternal-facing placenta samples in multiple adjusted linear regression analysis with EPDS at 26 weeks as the outcome. A significant linear correlation (rho = 0.843, p = 3e-129) is observed.


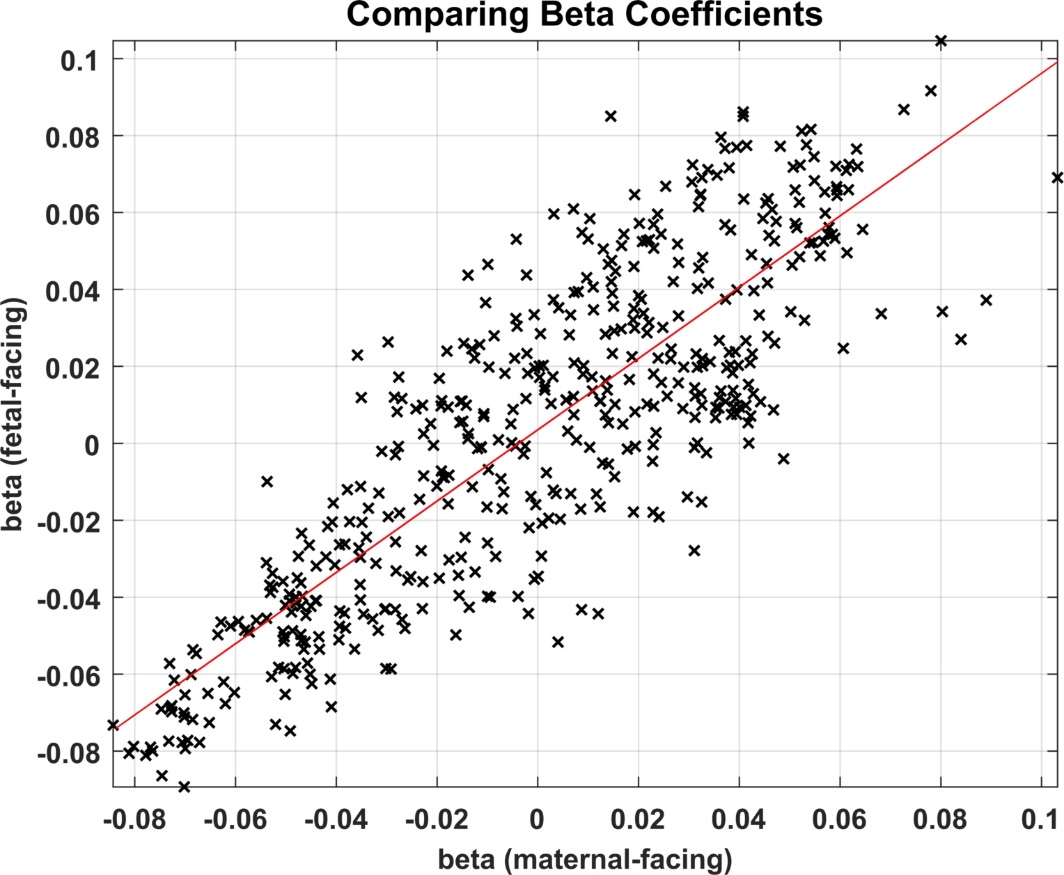


Figure S2. Coefficient of variation of all lipid species before batch-correction.

**
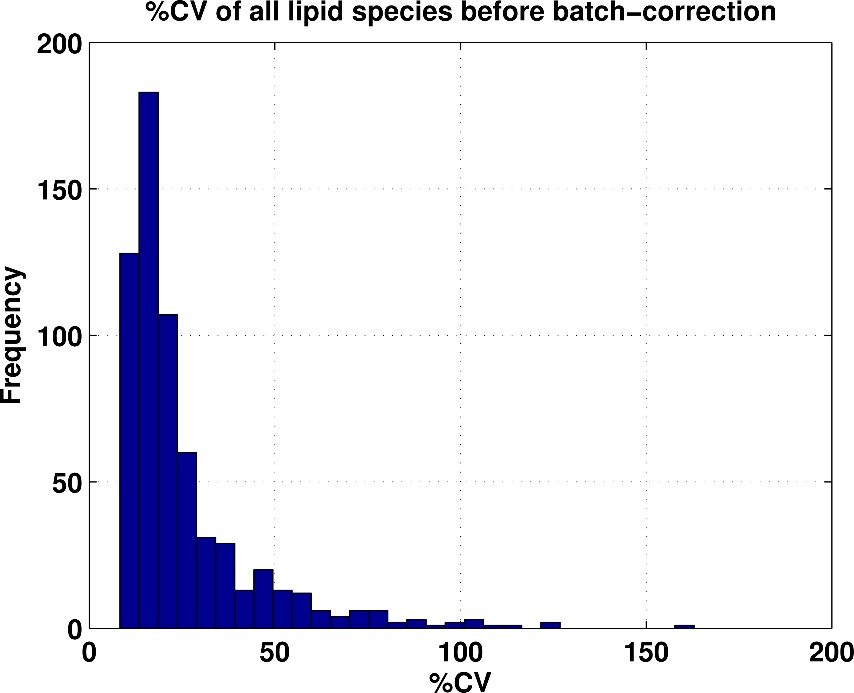
**


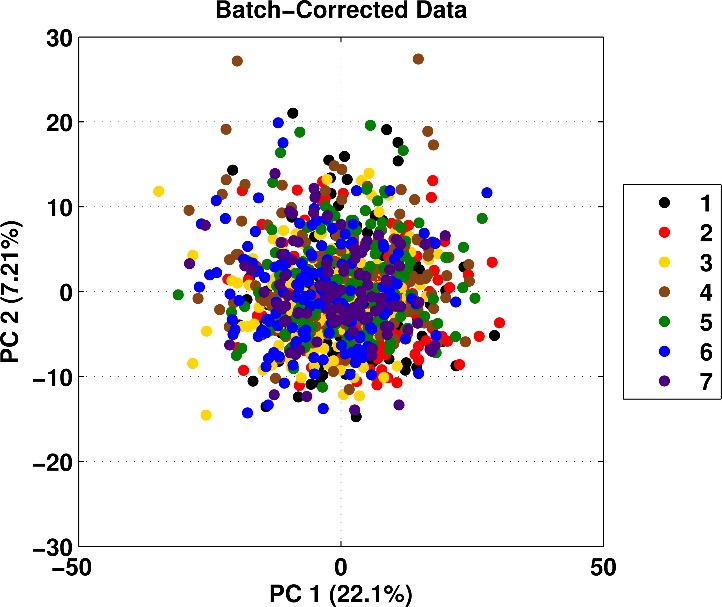

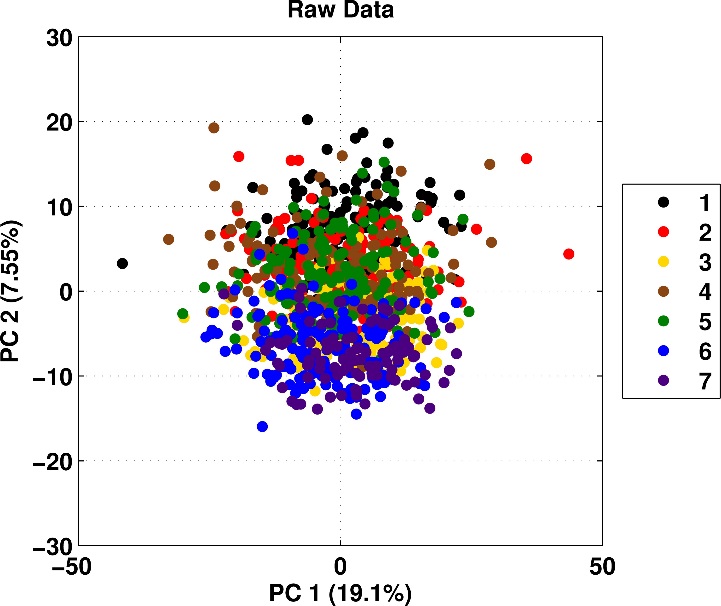
Figure S3. PCA of raw data and batch-corrected data.

Table S1. Characteristics of study subjects as classified by their Edinburgh Postnatal Depression Scale (EPDS) scores at 26 weeks.

| **Maternal Characteristics (mean)** | **EPDS (<2)** | **EPDS (> 10)** | **p** |
| --- | --- | --- | --- |
| Number of Subjects | 70 | 116 |  |
| Maternal Age (years) | 31·4 | 30·32 | 0·08 |
| Highest Education Age (years) | 21·5 | 19·3 | 0·0001 |
| Percentage of Chinese in Group | 80 | 72·4 | 0·25 |
| Percentage of Malay in Group | 20 | 27·6 |  |
| Parity | 0·81 | 1·02 | 0·13 |
| Pre-conception Maternal Weight (kg) | 55·6 | 56·3 | 0·79 |
| Maternal Height (m) | 1·59 | 1·58 | 0·14 |
| EPDS @ 26 weeks | 0·56 | 13·3 | 2E-90 |
| Percentage of gestational diabetes cases (as per WHO 1999 guidelines) | 20.29 | 19.64 | 1.00 |
| Percentage of preterm deliveries | 7.14 | 11.21 | 0.51 |
| Percentage with hypertensive disorders | 1.43 | 4.31 | 0.41 |
|  | | | |
| **Infant Characteristics (mean)** | **EPDS (<2)** | **EPDS (> 10)** | **p** |
| Gestational Age at Birth (days) | 273·8 | 270·1 | 0·009 |
| Birth Weight (kg) | 3·17 | 3·10 | 0·18 |
| Birth Length (cm) | 49·6 | 48·5 | 0·0005 |
| Head Circumference (cm) | 33·4 | 33·6 | 0·32 |

Table S2. Conditions for tandem mass spectrometry analysis of lipid species.

| Lipid class/subclass | Parent Ion | Fragmentation^*^ | Number of features | Internal standard | Internal standard (pmol) | Collision  Energy (V) |
| --- | --- | --- | --- | --- | --- | --- |
| Dihydroceramide (Cer(d18:0)) | [M+H]^+^ | NL, 18 Da | 6 | Cer(d18:0/8:0) | 50 | 21 |
| Ceramide (Cer(d18:1)) | [M+H]^+^ | PI, m/z 264·3 | 6 | Cer(d18:1/17:0) | 100 | 29 |
| Monohexocylceramide (HexCer) | [M+H]^+^ | PI, m/z 264·3 | 6 | Glucosylceramide 16:0 d3 | 50 | 33 |
| Dihexosylceramide (Hex2Cer) | [M+H]^+^ | PI, m/z 264·3 | 6 | Lactosylceramide 16:0 d3 | 50 | 53 |
| Trihexosylceramide (Hex3Cer) | [M+H]^+^ | PI, m/z 264·3 | 6 | Hex3Cer(17:0) | 50 | 57 |
| Sphingomyelin (SM) | [M+H]^+^ | PI, m/z 184·1 | 20 | SM(d18:1/12:0) | 200 | 25 |
| Phosphatidylcholine (PC) | [M+H]^+^ | PI, m/z 184·1 | 46 | PC(13:0/13:0) | 100 | 21 |
| Alkylphosphatidylcholine (PC(O)) | [M+H]^+^ | PI, m/z 184·1 | 19 | PC(13:0/13:0) | 100 | 21 |
| Alkenylphosphatidylcholine (PC(P)) | [M+H]^+^ | PI, m/z 184·1 | 14 | PC(13:0/13:0) | 100 | 21 |
| Lysophosphatidylcholine (LPC) | [M+H]^+^ | PI, m/z 184·1 | 22 | LPC(13:0) | 100 | 21 |
| Lysoalkylphosphatidylcholine (LPC(O)) | [M+H]^+^ | PI, m/z 104·1 | 10 | LPC(13:0) | 100 | 21 |
| Phosphatidylethanolamine (PE) | [M+H]^+^ | NL, 141 Da | 21 | PE(17:0/17:0) | 100 | 17 |
| Alkylphosphatidylethanolamine (PE(O)) | [M+H]^+^ | NL, 141 Da | 12 | PE(17:0/17:0) | 100 | 17 |
| Alkenylphosphatidylethanolamine (PE(P)) | [M+H]^+^ | NL, 141 Da | 11 | PE(17:0/17:0) | 100 | 17 |
| Lysophosphatidylethanolamine (LPE) | [M+H]^+^ | NL, 141 Da | 6 | LPE(14:0) | 100 | 17 |
| Phosphatidylinositol (PI) | [M+NH_4_]^+^ | NL, 277 Da | 16 | PE(17:0/17:0) | 100 | 17 |
| Lysophosphatidylinositol (LPI) | [M+NH_4_]^+^ | NL, 277 Da | 4 | PE(17:0/17:0) | 100 | 17 |
| Phosphatidylglycerol (PG) | [M+NH_4_]^+^ | NL, 189 Da | 3 | PG(17:0/17:0) | 100 | 21 |
| Cholesterol ester (CE) | [M+NH_4_]^+^ | PI, m/z 369·3 | 26 | CE(18:0)-d6 | 1000 | 10 |
| Free cholesterol (COH) | [M-H_2_O]^+^ | PI, m/z 161·2 | 1 | COH-d7 | 10000 | 23 |
| Diacylglycerol (DG) | [M+NH_4_]^+^ | NL, NH_3_ + fatty acid | 24 | DG(15:0/15:0) | 200 | 21 |
| Triacylglycerol (TG) | [M+NH_4_]^+^ | NL, NH_3_ + fatty acid | 25 | TG(17:0/17:0/17:0) | 100 | 5 |

^*^ PI, product ion; NL, neutral loss; SIM, single ion monitoring

| **Placental Lipids** | **Beta Coefficient** | **Lower Bound** | **Upper Bound** | **FDR-corrected p** |
| --- | --- | --- | --- | --- |
| PE(P-17:0/20:4)(a) | -0.585 | -0.873 | -0.298 | 0.036 |
| PE(P-17:0/22:6)(a) | -0.565 | -0.852 | -0.277 | 0.036 |
| PE(O-36:5) | -0.536 | -0.826 | -0.245 | 0.056 |
| PE(P-16:0/20:5) | -0.497 | -0.789 | -0.205 | 0.061 |
| PC 35:5 | -0.492 | -0.782 | -0.202 | 0.061 |
| PS 40:5 | -0.495 | -0.787 | -0.203 | 0.061 |
| PC 37:6 | -0.490 | -0.778 | -0.201 | 0.061 |
| PE 40:5(a) | -0.485 | -0.777 | -0.192 | 0.061 |
| PI 35:2 | -0.481 | -0.772 | -0.191 | 0.061 |
| PE(P-18:0/20:5) | -0.475 | -0.765 | -0.184 | 0.061 |
| PC 34:5 | -0.476 | -0.769 | -0.184 | 0.061 |
| PE(P-16:0/22:5)(a) | -0.477 | -0.769 | -0.184 | 0.061 |
| SM 35:1(b) | -0.465 | -0.756 | -0.174 | 0.063 |
| PC 33:2 | -0.459 | -0.747 | -0.170 | 0.063 |
| PC 39:5(a) | -0.460 | -0.752 | -0.168 | 0.063 |
| PI 40:5(a) | -0.459 | -0.752 | -0.167 | 0.063 |
| PE(P-19:0/20:4)(a) | -0.458 | -0.749 | -0.166 | 0.063 |
| PC(P-36:5) | -0.457 | -0.751 | -0.164 | 0.063 |

Table S3. List of placental lipids significantly associated (FDR p < 0.05) with EPDS at 26 weeks as determined by multiple adjusted linear regression without fish-oil supplementation as a covariate.

Table S4. Association of placental lipid levels with CBCL scales at 48 months in the offspring as determined by multiple adjusted linear regression without EPDS as a covariate (first 8 CBCL scales shown).

|  | **Emotionally Reactive** | | **Anxious/Depressed** | | **Somatic Complaints** | | **Withdrawn** | | **Internalizing Problems** | | **Attention Problems** | | **Aggressive Behaviour** | | **Externalizing Problems** | |
| --- | --- | --- | --- | --- | --- | --- | --- | --- | --- | --- | --- | --- | --- | --- | --- | --- |
|  | Beta | p | Beta | p | Beta | p | Beta | p | Beta | p | Beta | p | Beta | p | Beta | p |
| **PC 33:2** | -1.160 | 0.076 | **-1.420** | **0.034** | -0.423 | 0.546 | -1.449 | 0.083 | -1.877 | 0.095 | **-1.376** | **0.020** | -0.440 | 0.434 | -1.223 | 0.235 |
| **PC 34:5** | -1.241 | 0.059 | -1.230 | 0.069 | -1.322 | 0.059 | -1.150 | 0.173 | **-2.756** | **0.014** | -0.280 | 0.642 | -0.626 | 0.268 | -0.671 | 0.518 |
| **PC 35:5** | **-1.364** | **0.043** | **-1.367** | **0.049** | -1.289 | 0.073 | **-1.720** | **0.046** | **-2.646** | **0.022** | -0.948 | 0.123 | -0.963 | 0.096 | -1.671 | 0.116 |
| **PC 37:6** | **-1.362** | **0.036** | -1.195 | 0.074 | **-1.391** | **0.044** | -1.514 | 0.069 | **-2.345** | **0.036** | **-1.326** | **0.024** | -0.849 | 0.128 | -1.407 | 0.170 |
| **PC 39:5(a)** | **-1.381** | **0.034** | -1.180 | 0.079 | -1.254 | 0.071 | -1.154 | 0.168 | **-2.455** | **0.028** | **-1.343** | **0.023** | -0.815 | 0.145 | -1.479 | 0.150 |
| **PC(P-36:5)** | -0.749 | 0.244 | -0.618 | 0.351 | **-1.486** | **0.029** | -0.847 | 0.304 | -2.086 | 0.058 | -0.689 | 0.238 | -0.690 | 0.210 | -1.090 | 0.280 |
| **PE 40:5(a)** | **-1.778** | **0.006** | -1.190 | 0.077 | -1.110 | 0.111 | -1.368 | 0.102 | **-2.205** | **0.049** | **-1.887** | **0.001** | **-1.200** | **0.031** | -1.518 | 0.140 |
| PE(O-36:5) | 0.198 | 0.762 | 0.398 | 0.553 | 0.573 | 0.410 | 0.835 | 0.317 | 0.653 | 0.561 | -0.231 | 0.697 | 0.243 | 0.664 | 0.219 | 0.831 |
| **PE(P-16:0/20:5)** | **-1.499** | **0.020** | -0.868 | 0.193 | -1.025 | 0.137 | -0.634 | 0.446 | **-2.245** | **0.043** | **-1.507** | **0.010** | **-1.146** | **0.038** | -1.971 | 0.052 |
| **PE(P-16:0/22:5)(a)** | -1.169 | 0.065 | -0.778 | 0.235 | -0.737 | 0.277 | -0.254 | 0.756 | -1.703 | 0.119 | **-1.198** | **0.037** | -0.592 | 0.278 | -1.183 | 0.237 |
| **PE(P-17:0/20:4)(a)** | -0.859 | 0.201 | -0.750 | 0.279 | -1.152 | 0.107 | -1.570 | 0.067 | -2.102 | 0.068 | **-1.689** | **0.005** | -0.752 | 0.191 | **-2.380** | **0.023** |
| **PE(P-17:0/22:6)(a)** | -1.112 | 0.093 | -0.743 | 0.277 | -1.229 | 0.081 | -1.229 | 0.147 | -1.908 | 0.094 | **-1.504** | **0.012** | -0.959 | 0.090 | -1.950 | 0.060 |
| PE(P-18:0/20:5) | -1.063 | 0.102 | -0.738 | 0.271 | -0.792 | 0.254 | -0.898 | 0.281 | -1.709 | 0.126 | -0.736 | 0.214 | -0.647 | 0.246 | -1.329 | 0.193 |
| PI 35:2 | -0.588 | 0.363 | -0.979 | 0.140 | -0.545 | 0.429 | -0.587 | 0.478 | -1.162 | 0.296 | -0.951 | 0.104 | -0.400 | 0.470 | -1.022 | 0.314 |
| **PI 40:5(a)** | **-1.608** | **0.012** | **-1.394** | **0.035** | -1.081 | 0.116 | **-1.879** | **0.022** | **-2.691** | **0.015** | **-1.393** | **0.017** | **-1.100** | **0.046** | -1.530 | 0.132 |
| **PS 40:5** | -1.060 | 0.101 | -1.088 | 0.102 | **-1.495** | **0.029** | -0.957 | 0.249 | -2.066 | 0.063 | **-1.260** | **0.031** | -0.725 | 0.191 | -1.630 | 0.108 |
| **SM 35:1(b)** | -0.592 | 0.356 | **-1.385** | **0.034** | **-1.365** | **0.044** | -1.273 | 0.120 | **-2.274** | **0.038** | -0.787 | 0.176 | -0.596 | 0.277 | -1.098 | 0.275 |

Table S5. Association of placental lipid levels with CBCL scales at 48 months in the offspring as determined by multiple adjusted linear regression without EPDS as a covariate (last 7 CBCL scales shown).

|  | Sleep Problems | | DSM Affective Problems | | DSM Anxiety Problems | | DSM Pervasive Developmental Problems | | DSM ADHD | | DSM Oppositional Defiant Problems | | Total Problems | |
| --- | --- | --- | --- | --- | --- | --- | --- | --- | --- | --- | --- | --- | --- | --- |
|  | Beta | p | Beta | p | Beta | p | Beta | p | Beta | p | Beta | p | Beta | p |
| **PC 33:2** | -0.624 | 0.313 | **-1.497** | **0.037** | **-1.709** | **0.026** | -1.388 | 0.080 | -0.964 | 0.057 | -0.610 | 0.257 | -1.738 | 0.118 |
| **PC 34:5** | -0.889 | 0.153 | -1.238 | 0.088 | **-1.807** | **0.020** | **-1.970** | **0.013** | -0.239 | 0.642 | -0.342 | 0.528 | -2.012 | 0.072 |
| **PC 35:5** | -1.057 | 0.097 | **-1.483** | **0.046** | **-2.067** | **0.009** | **-2.153** | **0.008** | -0.733 | 0.163 | -0.727 | 0.191 | **-2.442** | **0.033** |
| **PC 37:6** | -1.159 | 0.058 | -1.274 | 0.076 | **-2.026** | **0.008** | **-1.772** | **0.024** | -0.972 | 0.054 | -0.659 | 0.218 | -2.094 | 0.058 |
| **PC 39:5(a)** | -1.087 | 0.077 | -1.106 | 0.125 | **-1.604** | **0.037** | -1.407 | 0.076 | **-1.086** | **0.031** | -0.859 | 0.109 | -2.163 | 0.051 |
| **PC(P-36:5)** | -0.516 | 0.395 | -0.447 | 0.530 | -1.126 | 0.138 | -1.317 | 0.091 | -0.515 | 0.303 | -0.134 | 0.800 | -1.741 | 0.110 |
| **PE 40:5(a)** | -0.787 | 0.203 | -1.014 | 0.161 | **-1.924** | **0.012** | -1.509 | 0.057 | -0.976 | 0.054 | -0.761 | 0.157 | -2.054 | 0.064 |
| PE(O-36:5) | 0.525 | 0.394 | 0.945 | 0.188 | 0.383 | 0.620 | 0.513 | 0.518 | 0.016 | 0.975 | 0.550 | 0.303 | 0.589 | 0.596 |
| **PE(P-16:0/20:5)** | -0.360 | 0.557 | -0.716 | 0.317 | -1.175 | 0.125 | -1.282 | 0.103 | **-1.030** | **0.040** | -0.860 | 0.105 | **-2.157** | **0.049** |
| **PE(P-16:0/22:5)(a)** | -0.610 | 0.310 | -0.407 | 0.563 | -1.354 | 0.071 | -0.547 | 0.480 | -0.488 | 0.324 | -0.480 | 0.358 | -1.568 | 0.147 |
| **PE(P-17:0/20:4)(a)** | -0.522 | 0.411 | -1.031 | 0.164 | -0.761 | 0.340 | **-1.852** | **0.022** | -0.837 | 0.108 | -0.792 | 0.151 | **-2.307** | **0.042** |
| **PE(P-17:0/22:6)(a)** | -1.097 | 0.079 | -0.986 | 0.178 | -1.237 | 0.115 | -1.497 | 0.062 | -0.935 | 0.069 | -0.529 | 0.332 | -2.064 | 0.066 |
| PE(P-18:0/20:5) | -0.635 | 0.301 | -0.518 | 0.472 | -1.298 | 0.091 | -1.308 | 0.097 | -0.529 | 0.296 | -0.216 | 0.687 | -1.700 | 0.124 |
| PI 35:2 | -0.301 | 0.621 | -0.220 | 0.758 | -1.157 | 0.130 | -0.765 | 0.330 | -0.696 | 0.165 | 0.088 | 0.868 | -1.232 | 0.262 |
| **PI 40:5(a)** | **-1.284** | **0.034** | **-1.514** | **0.033** | **-2.227** | **0.003** | **-1.908** | **0.014** | -0.960 | 0.055 | -0.833 | 0.116 | **-2.518** | **0.021** |
| **PS 40:5** | -0.668 | 0.275 | -0.505 | 0.481 | **-1.594** | **0.037** | -1.449 | 0.065 | -0.554 | 0.271 | -0.371 | 0.486 | **-2.232** | **0.042** |
| **SM 35:1(b)** | -0.491 | 0.417 | -1.139 | 0.106 | **-1.534** | **0.042** | -1.431 | 0.065 | **-1.043** | **0.035** | -0.696 | 0.185 | -1.847 | 0.089 |

**Experimental Methods**

***Placenta collection***

Five pieces of the placenta (of approximately 0.1 grams in weight) were biopsied at approximately 2 centimetres away from the umbilical cord insertion for the fetal-facing placenta (includes fetal chorionic plate and membranes with villous placenta) or its projection for the maternal facing placenta (includes maternal decidua with villous placenta). Tissue sections were rinsed quickly in Phosphate Buffer Saline solution (PBS). The tissues were sliced into smaller pieces and transferred into cryovials for immediate snap-freezing with liquid nitrogen before transfer to the -80°C freezer.

***LC-MS/MS lipid analysis***

A representative piece of placental tissue (50-100mg approximate. wet weight) was excised from the frozen sample and transferred to a pre-weighed “Safe Lock” Eppendorf tube (1.5mL). The sample was weighed, and one scoop of ZrO beads (1.0 mm diameter), and 1mL of water was added. Tissue was allowed to thaw, and groups of 24 were placed in a Bullet Blender and homogenised for 10 minutes, speed 11 at 4C. Homogenate was transferred into a fresh Eppendorf and immediately frozen at -80C. Homogenates were thawed, vortexed and sonicated by probe sonication (2 x 5 sec bursts, amplitude 25), before protein analysis using a Pierce BCA assay kit. Aliquots for lipid (20uL) and metabolite (50uL) analysis were taken at this time and frozen for subsequent extraction.

A detailed extraction protocol and analysis method was presented previously ^1,2^. Briefly, lipids were extracted from 20µL (approximately 100ug of protein) of placenta homogenate using chloroform/methanol (2:1, 20 volumes). Internal standards were added, and samples were vortexed and sonicated for 30 minutes. Following a 20 minute incubation, the samples were centrifuged and the supernatant transferred to 96 well plate and dried on a speedi-vac . Samples were reconstituted by the addition of 50uL of water saturated butanol, sonication (bath, 10 minutes) followed by 50uL MeOH with 10mM ammonium formate before transferring to 0.2mL micro-inserts in sample vials for analysis. Extractions were performed in 2 batches, each of which consisted of placenta samples, pooled biological placental quality control samples (every ten samples) and water blanks (every 20 samples).

***Processing and analysis of fatty acids in plasma phosphatidylcholine***

Maternal fasting blood samples collected during the 26-28 week clinic visit were processed within 4 hours and stored at -80^O^C thereafter. Dipentadecanoyl phosphatidylcholine was added to thawed plasma as internal standard prior to total lipid extraction with chloroform/methanol (2:1 vol/vol); butylated hydroxytoluene was added to the extraction as an antioxidant. Phosphatidylcholine (PC), which contributes about 75% of plasma phospholipid, was isolated by solid phase extraction on aminopropyl silica cartridges using chloroform to elute triacylglycerol and cholesteryl ester fractions, which were discarded, and then using chloroform/methanol (60:40 vol/vol) to elute the PC. Purified PC was dissolved in toluene and fatty acid methyl esters generated by reaction with methanol containing 2% (vol/vol) sulphuric acid at 50°C for 2 hours. After cooling and neutralisation, fatty acid methyl esters were extracted into hexane. Fatty acid methyl esters were separated by gas chromatography on a BPX-70 column (30 m × 220 μm; film thickness 0.25 μm) fitted to a Hewlett-Packard HP6890 gas chromatograph. Front inlet temperature was 300°C; initial column temperature was 115°C and was programmed to hold this temperature for 2 min, and then to increase temperature at 10°C/min to 200°C, to hold at 200°C for 10 min, to increase temperature at 10°C/min to 240°C, and then to hold this temperature for 2 min. Helium was used as the running gas, and fatty acid methyl esters were detected by flame ionisation. Fatty acid methyl esters were identified by comparison with retention times of standards run previously and they were quantified using the Aglient ChemStation software. The fatty acid concentration measurements are absolute and expressed as µg/mL of plasma.

**Statistical Methods**

1. Models 1 and 2 were used to study the association of placental and antenatal plasma lipids with EPDS, respectively. EPDS and fish-oil supplementation were used as categorical variables, while lipids and BMI were used as continuous measures. We adjusted for the confounding effects of fish-oil supplementation and ppBMI in these models as both the factors are known to modulate lipid levels in circulation.^3, 4^

- **Model 1:** placental lipid levels ~ fish-oil supplementation + pre-pregnancy BMI + β_1_EPDS at 26 weeks
- **Model 2:** fatty acid levels ~ fish-oil supplementation + pre-pregnancy BMI + β_2_EPDS at 26 weeks

1. Models 3 and 4 were used to study the downstream effects of antenatal lipids and maternal mental health on child CBCL scores. The covariates used were gestational age at delivery, infant gender and maternal education (a surrogate variable for socio-economic status). Maternal SES and child gender were selected as covariates as these are known to influence the child CBCL scores^5,6,7^. We also adjusted these models for gestational age as it is (1) related to fetal brain development, and (2) was significantly different between the two EPDS groups (Table S1).

- **Model 3:** CBCL scales at 48 months ~ maternal education + gestational age at delivery + infant gender + β_3_EPDS at 26 weeks
- **Model 4:** CBCL scales at 48 months ~ maternal education + gestational age at delivery + infant gender + EPDS at 26 weeks + β_4_placental lipid levels

**References**

1. Weir JM, *et al.* Plasma lipid profiling in a large population-based cohort. *Journal of lipid research* 2013; **54**(10): 2898-908.

2. Alshehry ZH, *et al*. An Efficient Single Phase Method for the Extraction of Plasma Lipids. *Metabolites* 2015; **5**(2): 389-403.

3. Farias DR, *et al.* Lipid changes throughout pregnancy according to pre-pregnancy BMI: results from a prospective cohort. *BJOG : an international journal of obstetrics and gynaecology* 2016; **123**(4): 570-8.

4. Dunstan JA, *et al*. The Effects of Fish Oil Supplementation in Pregnancy on Breast Milk Fatty Acid Composition Over the Course of Lactation: A Randomized Controlled Trial. *Pediatric Research* 2007; **62**(6): 689-94.

5. Hosokawa R, Katsura T. Effect of socioeconomic status on behavioral problems from preschool to early elementary school - A Japanese longitudinal study. *PLoS One* 2018; **13**(5): e0197961-e.

6. Neumann CS, Grimes K, Walker EF, Baum K. Developmental pathways to schizophrenia: Behavioral subtypes. *Journal of Abnormal Psychology* 1995; **104**(4): 558-66.

7. Walker E, *et al.* Search for the Causes of Schizophrenia. 1995.
